# Association of the triglyceride–cholesterol–body weight index (TCBI) with incident diabetes in Chinese adults: a population-based cohort study with non-linear and threshold analyses

**DOI:** 10.3389/fnut.2026.1928555

**Published:** 2026-09-30

**Authors:** Xue Guo, Yao Wu, Guangqin Ren, Huibing Chen, Yang Song, Lijuan Dong

**Affiliations:** 1 The Tenth Clinical Medical College of Guangzhou University of Traditional Chinese Medicine, Zhongshan, China; 2 Zhongshan Hospital of Traditional Chinese Medicine, Zhongshan, China; 3 Guangzhou University of Chinese Medicine, Guangzhou, China

**Keywords:** Chinese adults, cohort study, incident diabetes, non-linear relationship, threshold effect, triglyceride–cholesterol–body weight index

## Abstract

**Background:**

The triglyceride–cholesterol–body weight index (TCBI) is a simple composite nutritional marker. However, its association with incident diabetes remains unclear. We examined this association in Chinese adults, focusing on non-linear and threshold effects.

**Methods:**

This retrospective cohort study included 206,085 adults without baseline diabetes. TCBI was calculated as (TG × TC × body weight) / 1,000. We used Cox regression, restricted cubic splines, and segmented regression for analysis.

**Results:**

During follow-up, 4,093 participants developed incident diabetes. Higher TCBI was consistently associated with an increased risk of incident diabetes. In the fully adjusted model, each 1-SD increase in TCBI was associated with a 13% higher risk of incident diabetes (HR = 1.13, 95% CI: 1.12–1.14; *P* < 0.001), while each 1-unit increase in ln-TCBI was associated with a 94% higher risk (HR = 1.94, 95% CI: 1.86–2.03; *P* < 0.001). Compared with participants in the lowest TCBI quartile (Q1), the fully adjusted HRs were 2.18 (95% CI: 1.83–2.59), 3.49 (95% CI: 2.95–4.12), and 6.53 (95% CI: 5.53–7.72) for Q2, Q3, and Q4, respectively (*P* for trend < 0.001). Restricted cubic spline analysis indicated a non-linear association between ln-TCBI and incident diabetes (P for non-linearity < 0.001). Segmented regression identified a potential threshold at ln-TCBI = 6.828 (original TCBI ≈ 923), with positive associations observed both below (HR = 2.223, 95% CI: 1.613–3.062) and above (HR = 1.901, 95% CI: 1.787–2.022) the threshold.

**Conclusions:**

These findings suggest that TCBI may represent an accessible composite metabolic indicator associated with diabetes risk; however, whether TCBI provides additional information beyond established metabolic indicators and whether the identified threshold has clinical relevance require further validation in diverse populations.

## 1 Introduction

Type 2 diabetes mellitus (T2DM) has emerged as one of the most pressing public health challenges worldwide. The Global Burden of Disease Study 2021 estimated that approximately 529 million people were living with diabetes globally in 2021, with this number projected to exceed 1.3 billion by 2050 (1). Consistent with this substantial disease burden, the International Diabetes Federation has documented a continuing increase in the global prevalence and economic burden of diabetes (2, 3). China accounts for a substantial proportion of this burden, with national epidemiological studies showing a marked increase in diabetes prevalence among Chinese adults over recent decades (4, 5), highlighting the growing need for effective and integrated strategies for diabetes prevention and care (6). Of particular concern is the increasing burden of diabetes among younger adults in China (7). Early-onset T2DM is associated with longer cumulative exposure to hyperglycemia and substantially higher risks of cardiovascular complications and premature mortality than later-onset disease (8, 9). These trends underscore the importance of identifying readily available metabolic indicators associated with diabetes risk to facilitate early risk identification and inform preventive strategies.

Excess adiposity represents a well-established and potent risk factor for T2DM. A meta-analysis synthesizing 18 prospective cohort studies found that obesity conferred a sevenfold elevation in diabetes risk relative to normal weight (10). Accumulating evidence further indicates that the metabolic consequences of excess adiposity are especially detrimental in younger populations (11). Nevertheless, the widely adopted body mass index (BMI) has notable shortcomings as a surrogate for adiposity-related risk. BMI fails to differentiate fat mass from lean mass, provides no information on fat distribution, and does not incorporate metabolic parameters (12, 13). Visceral adipose tissue—which exhibits a stronger correlation with insulin resistance and cardiometabolic disease than subcutaneous fat—remains unaccounted for in BMI (14). These limitations underscore the need for more integrative indices that combine anthropometric and metabolic measures to better characterize cardiometabolic risk.

The triglyceride–cholesterol–body weight index (TCBI)—calculated as (TG × TC × body weight) / 1,000, with TG and TC expressed in mg/dL—was initially introduced as a simple prognostic marker in patients with coronary artery disease (15). Other composite metabolic indices, such as the triglyceride-glucose-body mass index (TyG-BMI), have also been increasingly investigated in relation to diabetes and cardiovascular outcomes (16). More recently, TCBI has been examined in other clinical contexts, including nephrolithiasis and cardiovascular disease (17). Nevertheless, evidence regarding the association between TCBI and incident diabetes in the general population remains limited. In particular, the dose–response pattern, potential threshold characteristics, and potential population heterogeneity of this association warrant further investigation.

Previous investigations using the same cohort have shown that the association between BMI and incident diabetes is more pronounced in younger adults (18). Age- and sex-related heterogeneity in diabetes risk has also been reported across Asian populations (19, 20). Whether TCBI—a composite measure incorporating lipid and body-weight information—shows similar population heterogeneity warrants further investigation. Accordingly, we conducted a large retrospective cohort study of Chinese adults to: (1) evaluate the association between TCBI and incident diabetes; (2) characterize its dose–response pattern and potential threshold using restricted cubic spline and segmented regression analyses; and (3) examine potential effect modification across key demographic and lifestyle subgroups.

## 2 Methods

### 2.1 Study design and population

This retrospective cohort study used data from a computerized health screening database. The database was established by the Rich Healthcare Group in China. It contains medical records of individuals who underwent annual health evaluations between 2010 and 2016. The study protocol was approved by the Rich Healthcare Group Review Board. All participant data were anonymized prior to analysis.

The parent cohort included 211,833 participants aged 20 years or older. All had completed at least two health check visits. Detailed exclusion criteria have been reported elsewhere (18). For the current investigation, we excluded individuals with incomplete TCBI data. Specifically, 4,854 participants had missing total cholesterol. Another 34 had missing triglyceride. We also excluded 860 participants with triglyceride values of zero. Log-transformation required for statistical modeling necessitated this exclusion. The final analytical cohort comprised 206,085 participants (Figure 1).

**Figure 1:**
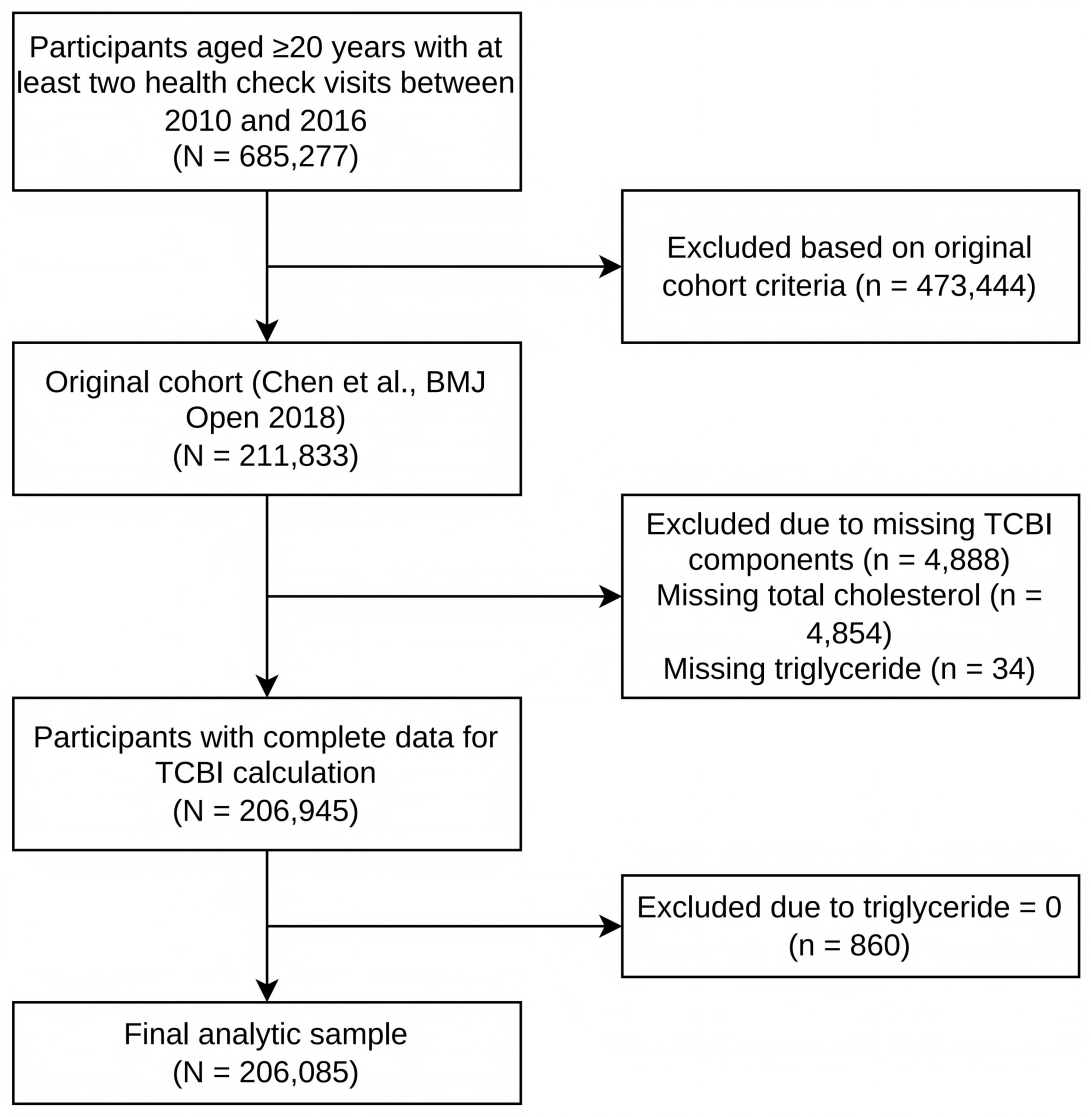
Participant selection flowchart.

Initially, 685,277 participants aged ≥20 years with at least two health check visits (2010–2016) were screened. After applying exclusion criteria from the original study (15), 211,833 participants formed the parent cohort. For the present analysis, we excluded those with missing total cholesterol (*n* = 4,854) or missing triglyceride (*n* = 34). We also excluded 860 participants with triglyceride values of zero (required for log-transformation). The final sample included 206,085 participants. TCBI, triglyceride–total cholesterol–body weight index.

### 2.2 Data collection and definitions

At each visit, participants completed a structured questionnaire. The questionnaire captured demographic information, lifestyle behaviors (smoking and alcohol consumption), and medical history. Family history of diabetes received particular attention.

Trained personnel performed anthropometric measurements following standardized protocols. Body weight was recorded to the nearest 0.1 kg. Participants wore light clothing and no footwear. Height was measured to the nearest 0.1 cm. BMI was calculated as weight in kilograms divided by height in meters squared. Blood pressure was measured using standard mercury sphygmomanometers.

Fasting venous blood samples were collected after an overnight fast of at least 10 h. Serum concentrations of total cholesterol (TC), triglyceride (TG), high-density lipoprotein cholesterol (HDL-c), and low-density lipoprotein cholesterol (LDL-c) were quantified. Alanine aminotransferase (ALT), aspartate aminotransferase (AST), blood urea nitrogen (BUN), and creatinine clearance rate (CCR) were also measured. All analyses were performed using an autoanalyzer (Beckman 5800). Fasting plasma glucose (FPG) was measured using the glucose oxidase method on the same platform.

### 2.3 Calculation of TCBI

TCBI was calculated using a previously established formula (15):


$$\begin{array}{l} 6\text{TCBI}=\frac{\left({\text{TG}}_{mg/dL}\times {\text{TC}}_{mg/dL}\times {\text{bodyweight}}_{kg}\right)}{1000} \end{array}$$

TG and TC were converted from mmol/L to mg/dL. Conversion factors of 88.57 and 38.67 were applied, respectively.

TCBI was operationalized in three forms for analysis. First, it was used as a continuous variable after natural logarithmic transformation (ln-TCBI). This transformation approximated normality. Second, it was categorized into quartiles (Q1 to Q4) based on the distribution of original TCBI values. Third, it was standardized as a Z-score variable for sensitivity analyses.

### 2.4 Ascertainment of incident diabetes

Incident diabetes was ascertained using criteria consistent with the parent study. A follow-up FPG measurement of 7.0 mmol/L or higher indicated diabetes. A self-reported clinician diagnosis of diabetes during follow-up also qualified. Participants were censored at the earliest of three dates: diabetes diagnosis, last follow-up visit, or study termination.

### 2.5 Statistical analysis

All statistical analyses were performed using R software (version 3.3.2; R Foundation for Statistical Computing, Vienna, Austria) and Free Statistics software (version 2.4.0; Beijing, China). Statistical significance was set at a two-sided *P* < 0.05.

### 2.6 Baseline characteristics

Participants were divided into four groups based on ascending TCBI quartiles. Baseline characteristics were summarized as mean ± standard deviation (SD) for normally distributed continuous variables. Skewed variables (TG and ALT) were presented as median (interquartile range, IQR). Categorical variables were reported as frequency (percentage). Between-group comparisons used one-way ANOVA for normally distributed variables. The Kruskal–Wallis test was applied to skewed variables. The chi-square test was used for categorical variables.

### 2.7 Survival analysis

Kaplan–Meier survival curves depicted diabetes-free survival across TCBI quartiles. Between-group differences were assessed using the log-rank test.

Cox proportional hazards regression models were used to estimate hazard ratios (HRs) and 95% confidence intervals (CIs) for the association between TCBI and incident diabetes. Potential confounders were selected based on clinical relevance and previous studies (21). Four sequential Cox proportional hazards regression models were constructed. The crude model was unadjusted. Model I was adjusted for age and sex. Model II was additionally adjusted for systolic blood pressure (SBP), diastolic blood pressure (DBP), family history of diabetes, smoking status, and drinking status. Model III was further adjusted for alanine aminotransferase (ALT), aspartate aminotransferase (AST), blood urea nitrogen (BUN), and creatinine clearance rate (CCR).

TCBI was examined in three forms: (1) ln-transformed continuous variable; (2) quartiles (Q1 as reference); and (3) standardized continuous variable (per 1-SD increase) for sensitivity analyses.

### 2.8 Assessment of non-linearity and threshold effects

We used restricted cubic splines (RCS) to characterize the shape of the ln-TCBI–diabetes relationship. Three knots were placed at the 10th, 50th, and 90th percentiles of the ln-TCBI distribution. The Wald test assessed overall association and non-linearity.

We then fitted a segmented (two-piece) Cox regression model. This identified the optimal breakpoint (threshold) of ln-TCBI. The model estimated separate slopes for segments below and above the breakpoint. A likelihood ratio test compared the two-piece model with a standard linear model.

### 2.9 Subgroup analyses

Predefined subgroup analyses were performed to assess the consistency of the association between TCBI and incident diabetes across strata of age (< 65 vs. ≥65 years), sex (male vs. female), family history of diabetes (yes vs. no), smoking status (current, former, never), and drinking status (current, former, never). For each subgroup, the fully adjusted Cox model (Model III) was refitted to estimate HRs for ln-TCBI. Effect modification was assessed by including multiplicative interaction terms between ln-TCBI and each subgroup variable in the Cox models, with *P* values for interaction derived from likelihood ratio tests comparing models with and without the interaction term. Given that five interaction tests were performed, a Bonferroni correction was applied to account for multiple comparisons, with a corrected significance threshold of *P* < 0.01 (0.05/5) for interaction tests. The results were presented using a forest plot.

### 2.10 Sensitivity analyses

We conducted several sensitivity analyses to test the robustness of our findings. First, we repeated the primary analyses using TCBI as a standardized continuous variable (per 1-SD increase). Second, we examined the association using quartile-based categorization. Third, we reassessed the threshold effect using log10-transformed TCBI. This confirmed the stability of the breakpoint estimate.

## 3 Results

### 3.1 Study population and baseline characteristics

This analysis included 206,085 participants with complete TCBI data. Mean age was 42.2 (SD 12.7) years. Male participants comprised 54.8% of the cohort.

Participants were stratified into quartiles based on TCBI distribution (Q1 to Q4, ascending). Table 1 summarizes baseline characteristics across quartiles. A stepwise deterioration in metabolic profiles was observed with increasing TCBI.

**Table 1:** Baseline characteristics by TCBI quartiles.

Characteristics	Total (*N* = 206,085)	Q1 (*n* = 50,876)	Q2 (*n* = 51,736)	Q3 (*n* = 51,736)	Q4 (*n* = 51,737)	*P*-value
Demographic						
Age (years), Mean ± SD	42.2 ± 12.7	36.9 ± 9.8	40.9 ± 12.2	44.7 ± 13.4	46.3 ± 12.9	< 0.001
Male, *n* (%)	113,005 (54.8)	14,154 (27.8)	24,250 (46.9)	33,371 (64.5)	41,230 (79.7)	< 0.001
Anthropometric						
Height (cm), Mean ± SD	166.4 ± 8.3	163.2 ± 7.5	165.6 ± 8.1	167.4 ± 8.3	169.5 ± 8.0	< 0.001
Weight (kg), Mean ± SD	64.7 ± 12.2	55.4 ± 8.0	61.3 ± 9.5	67.3 ± 10.3	74.7 ± 11.4	< 0.001
BMI (kg/m^2^), Mean ± SD	23.2 ± 3.3	20.7 ± 2.3	22.3 ± 2.7	24.0 ± 2.8	25.9 ± 3.0	< 0.001
Blood pressure						
SBP (mmHg), Mean ± SD	119.1 ± 16.4	111.8 ± 13.8	116.6 ± 15.3	121.6 ± 16.2	126.3 ± 16.5	< 0.001
DBP (mmHg), Mean ± SD	74.2 ± 10.8	69.5 ± 9.4	72.4 ± 10.0	75.5 ± 10.4	79.2 ± 11.0	< 0.001
Glycemic						
Baseline FPG (mmol/L), Mean ± SD	4.9 ± 0.6	4.8 ± 0.5	4.9 ± 0.6	5.0 ± 0.6	5.1 ± 0.6	< 0.001
Lipid profile						
TC (mmol/L), Mean ± SD	4.7 ± 0.9	4.1 ± 0.6	4.5 ± 0.7	4.9 ± 0.8	5.4 ± 0.9	< 0.001
TG (mmol/L), Median (IQR)	1.1 (0.7–1.6)	0.6 (0.5–0.7)	0.9 (0.8–1.0)	1.3 (1.1–1.5)	2.2 (1.8–2.8)	< 0.001
HDL-c (mmol/L), Mean ± SD	1.4 ± 0.3	1.4 ± 0.2	1.4 ± 0.3	1.4 ± 0.3	1.3 ± 0.3	< 0.001
LDL-c (mmol/L), Mean ± SD	2.7 ± 0.7	2.3 ± 0.5	2.6 ± 0.5	2.9 ± 0.6	3.1 ± 0.7	< 0.001
Liver function						
ALT (U/L), Median (IQR)	18.0 (13.0–27.5)	13.0 (10.5–17.9)	15.8 (12.0–22.0)	20.0 (14.6–28.5)	27.9 (19.4–41.5)	< 0.001
AST (U/L), Mean ± SD	24.0 ± 11.0	21.1 ± 8.5	22.5 ± 11.3	24.4 ± 10.4	28.1 ± 12.0	< 0.001
Renal function						
BUN (mmol/L), Mean ± SD	4.7 ± 1.1	4.5 ± 1.1	4.6 ± 1.2	4.8 ± 1.2	4.8 ± 1.1	< 0.001
CCR (umol/L), Mean ± SD	70.0 ± 15.7	63.1 ± 14.1	68.1 ± 15.3	72.5 ± 15.5	76.1 ± 14.8	< 0.001
Lifestyle						
Current smoker, *n* (%)	32,421 (15.7)	2,517 (4.9)	5,351 (10.3)	9,187 (17.8)	15,366 (29.7)	< 0.001
Current drinker, *n* (%)	1,946 (0.9)	135 (0.3)	301 (0.6)	535 (1.0)	975 (1.9)	< 0.001
Family history						
Family history of diabetes, *n* (%)	4,223 (2.0)	946 (1.9)	1,104 (2.1)	1,068 (2.1)	1,105 (2.1)	0.005
Follow-up outcomes						
Incident diabetes, *n* (%)	4,093 (2.0)	176 (0.3)	531 (1.0)	1,070 (2.1)	2,316 (4.5)	< 0.001
Follow-up duration (years), Mean ± SD	3.1 ± 0.9	3.2 ± 1.0	3.1 ± 0.9	3.1 ± 0.9	3.1 ± 0.9	< 0.001

Data are mean ± SD, median (IQR), or *n* (%). TCBI was calculated as (TG [mg/dL] × TC [mg/dL] × body weight [kg]) / 1,000. TG and TC were converted from mmol/L to mg/dL using factors of 88.57 and 38.67, respectively. Q1 to Q4 denote ascending quartiles. TCBI, triglyceride–total cholesterol–body weight index; SD, standard deviation; IQR, interquartile range; BMI, body mass index; SBP, systolic blood pressure; DBP, diastolic blood pressure; FPG, fasting plasma glucose; TC, total cholesterol; TG, triglyceride; HDL-c, high-density lipoprotein cholesterol; LDL-c, low-density lipoprotein cholesterol; ALT, alanine aminotransferase; AST, aspartate aminotransferase; BUN, blood urea nitrogen; CCR, creatinine clearance rate.

Participants in the highest quartile (Q4) were older than those in the lowest (Q1) (46.3 vs. 36.9 years). They were more frequently male (79.7% vs. 27.8%). They had substantially higher BMI (25.9 vs. 20.7 kg/m^2^), elevated blood pressure, and higher fasting glucose. Their lipid profile was more atherogenic, with increased total cholesterol, triglycerides, and LDL-cholesterol, and reduced HDL-cholesterol.

Higher TCBI quartiles also correlated with elevated liver enzymes (ALT, AST). Current smoking and alcohol consumption were more prevalent in higher quartiles.

Cumulative diabetes incidence rose progressively from 0.3% in Q1 to 4.5% in Q4 (*P* for trend < 0.001). Median follow-up duration was comparable across groups at approximately 3.1 years.

### 3.2 TCBI and risk of incident diabetes

During a median follow-up of 3.1 years (643,954 person-years), we documented 4,093 incident diabetes cases. The overall incidence rate was 6.36 per 1,000 person-years.

Kaplan–Meier survival curves showed early and sustained separation across TCBI quartiles. Higher quartile groups had significantly lower diabetes-free survival (log-rank *P* < 0.001, Figure 2).

**Figure 2:**
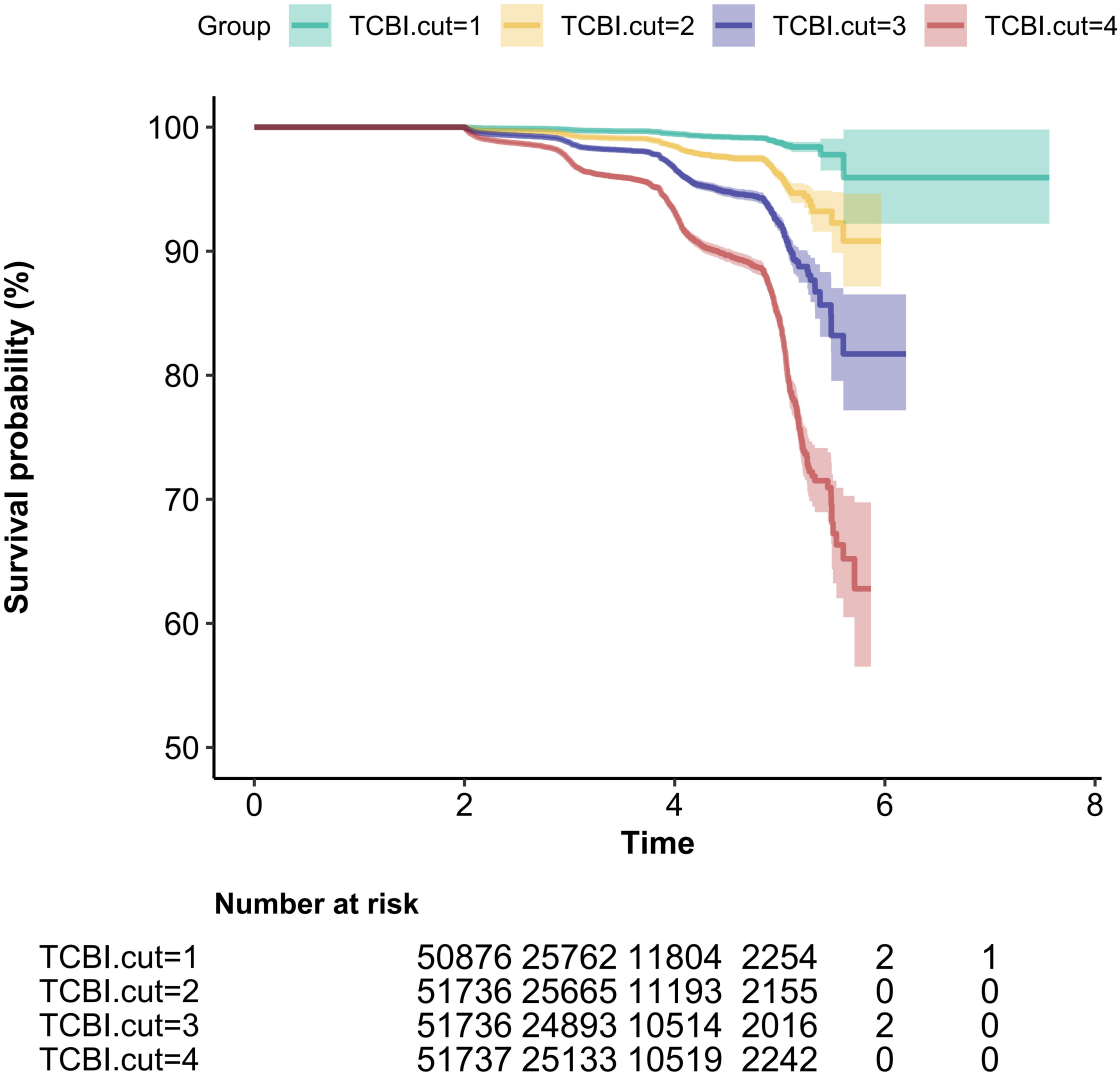
Kaplan–Meier survival curves by TCBI quartiles.

Cumulative diabetes-free survival over a median follow-up of 3.1 years among 206,085 participants. Curves are stratified by TCBI quartiles (Q1 to Q4, lowest to highest). Shaded areas indicate 95% confidence intervals. The log-rank test showed significant differences across groups (*P* < 0.001). TCBI was calculated as (TG [mg/dL] × TC [mg/dL] × body weight [kg]) / 1,000. TG and TC were converted from mmol/L to mg/dL using factors of 88.57 and 38.67, respectively. Abbreviations: TCBI, triglyceride–total cholesterol–body weight index.

Table 2 shows the association between TCBI and incident diabetes across the sequential Cox regression models. Higher TCBI was consistently associated with an increased risk of incident diabetes in all models. In the fully adjusted Model III, each 1-SD increase in TCBI was associated with a 13% higher risk of incident diabetes (HR = 1.13, 95% CI: 1.12–1.14, *P* < 0.001). When TCBI was log-transformed, the associations remained significant, with HRs of 4.61 (95% CI: 4.19–5.08, *P* < 0.001) for log10-TCBI and 1.94 (95% CI: 1.86–2.03, *P* < 0.001) for ln-TCBI.

**Table 2:** Multivariable Cox regression for TCBI and incident diabetes.

Variable	Crude model		Model I		Model II		Model III	
	HR (95% CI)	*P*	HR (95% CI)	*P*	HR (95% CI)	*P*	HR (95% CI)	*P*
TCBI (per 1 SD increase)	1.15 (1.14~1.15)	< 0.001	1.15 (1.14~1.15)	< 0.001	1.14 (1.13~1.15)	< 0.001	1.13 (1.12~1.14)	< 0.001
log10-TCBI	9.21 (8.5~9.98)	< 0.001	6.19 (5.64~6.79)	< 0.001	4.92 (4.46~5.42)	< 0.001	4.61 (4.19~5.08)	< 0.001
ln-TCBI	2.62 (2.53~2.72)	< 0.001	2.21 (2.12~2.3)	< 0.001	2.00 (1.91~2.08)	< 0.001	1.94 (1.86~2.03)	< 0.001
TCBI quartiles								
Q1	1.00 (Ref)		1.00 (Ref)		1.00 (Ref)		1.00 (Ref)	
Q2	3.07 (2.58~3.63)	< 0.001	2.15 (1.81~2.55)	< 0.001	2.05 (1.73~2.43)	< 0.001	2.18 (1.83–2.59)	< 0.001
Q3	6.42 (5.48~7.53)	< 0.001	3.47 (2.95~4.08)	< 0.001	3.11 (2.64~3.66)	< 0.001	3.49 (2.95–4.12)	< 0.001
Q4	13.65 (11.71~15.91)	< 0.001	6.65 (5.68~7.79)	< 0.001	5.43 (4.63~6.37)	< 0.001	6.53 (5.53–7.72)	< 0.001
*P* for trend		< 0.001		< 0.001		< 0.001		< 0.001

Crude model: unadjusted. Model I: adjusted for age and sex. Model II: additionally adjusted for SBP, DBP, family history of diabetes, smoking status, and drinking status. Model III: additionally adjusted for ALT, AST, BUN, and CCR. TCBI was analyzed as a standardized variable (per 1 SD), log10-transformed, ln-transformed, and as quartiles. Abbreviations: HR, hazard ratio; CI, confidence interval; SD, standard deviation. For other abbreviations, see Table 1 footnote.

When TCBI was analyzed by quartiles, the risk of incident diabetes increased progressively across higher TCBI quartiles. Compared with participants in Q1, the fully adjusted HRs were 2.18 (95% CI: 1.83–2.59) for Q2, 3.49 (95% CI: 2.95–4.12) for Q3, and 6.53 (95% CI: 5.53–7.72) for Q4 (all P < 0.001). The trend across TCBI quartiles was statistically significant (*P* for trend < 0.001). Univariate Cox regression analyses for each potential risk factor are presented in Supplementary Table 1.

### 3.3 Non-linear dose-response relationship

We fitted restricted cubic splines to further characterize this relationship. The analysis revealed a significant non-linear association between ln-TCBI and diabetes risk (*P* for overall < 0.001; *P* for non-linearity < 0.001) (Figure 3).

**Figure 3:**
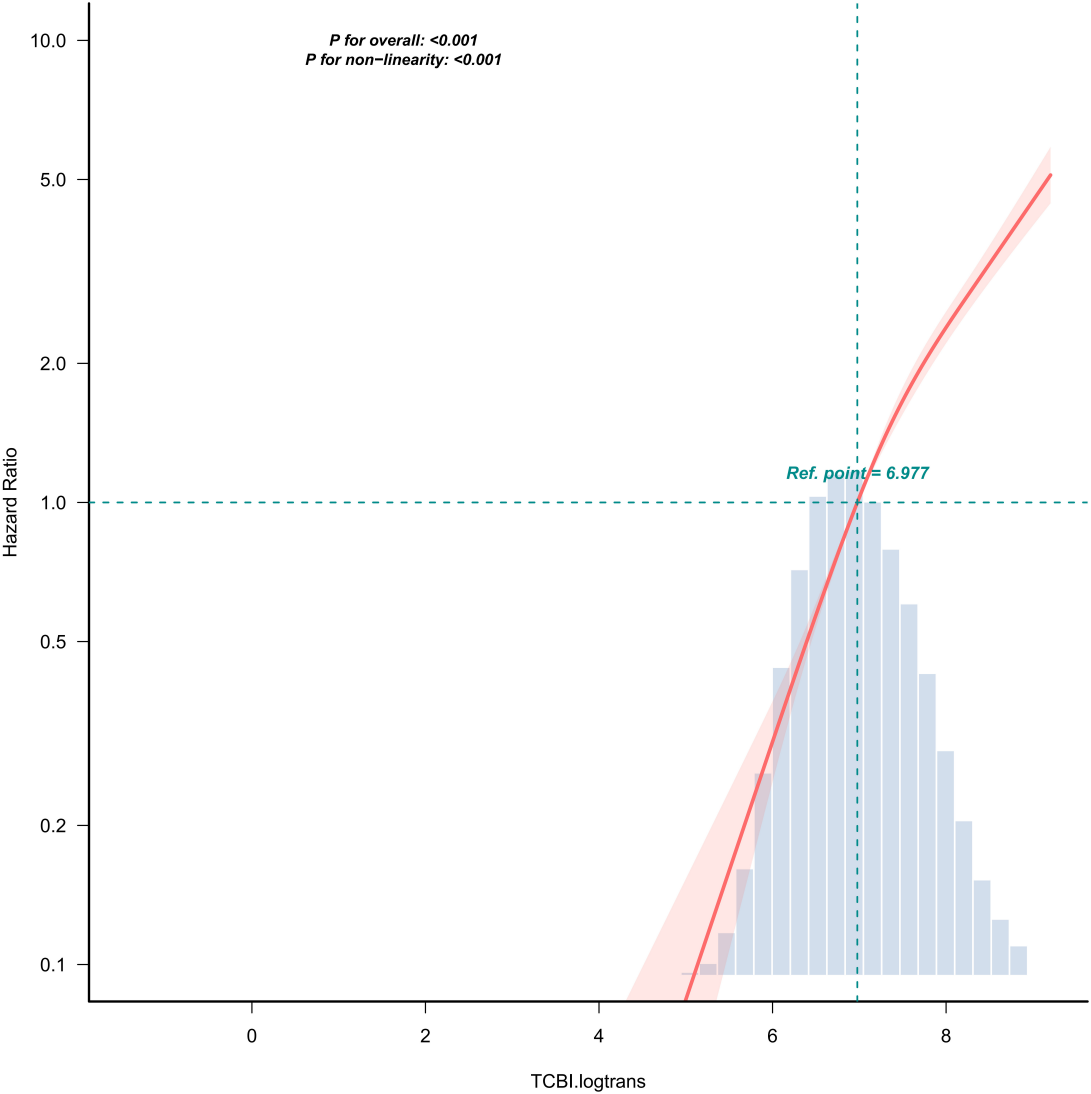
Restricted cubic spline for ln-TCBI and incident diabetes.

Segmented Cox regression identified a potential threshold at ln-TCBI = 6.828, corresponding to an original TCBI value of approximately 923. Below the threshold, higher ln-TCBI was associated with an increased risk of incident diabetes (HR = 2.223, 95% CI: 1.613–3.062), while above the threshold, the association remained positive (HR = 1.901, 95% CI: 1.787–2.022). The two-piece model provided a better fit than the single-line model (likelihood ratio test, *P* = 0.003) (Table 3).

**Table 3:** Threshold effect of ln-transformed TCBI on incident diabetes.

TCBI level	HR (95% CI)	*P*-value
ln-TCBI < 6.828 (original TCBI < 923)	2.223 (1.613, 3.062)	< 0.001
ln-TCBI ≥ 6.828 (original TCBI ≥ 923)	1.901 (1.787, 2.022)	< 0.001
Likelihood Ratio test	-	0.003

Adjusted for age, sex, SBP, DBP, family history of diabetes, smoking, drinking, ALT, AST, BUN, and CCR. Breakpoint: ln-TCBI = 6.828 (original TCBI ≈ 923). Likelihood ratio test: two-piece model vs. linear model.

Adjusted for age, sex, SBP, DBP, family history of diabetes, smoking, drinking, ALT, AST, BUN, and CCR. Solid line: hazard ratio. Shaded area: 95% confidence interval. Bottom histogram: distribution of ln-TCBI. *P* for overall < 0.001; P for non-linearity < 0.001. TCBI, triglyceride–total cholesterol–body weight index.

### 3.4 Subgroup analyses

Subgroup analyses showed significant interactions of ln-TCBI with age, sex, and smoking status (all *P* for interaction < 0.001), which remained significant after Bonferroni correction for five interaction tests (corrected significance threshold, *P* < 0.01). The association was stronger among participants aged < 65 years (HR = 1.97, 95% CI: 1.88–2.07) than among those aged ≥65 years (HR = 1.29, 95% CI: 1.16–1.44), and among women (HR = 2.17, 95% CI: 2.00–2.37) than among men (HR = 1.80, 95% CI: 1.71–1.89). Among smoking subgroups, the HRs were 1.70 (95% CI: 1.59–1.82), 2.58 (95% CI: 2.04–3.28), and 2.05 (95% CI: 1.94–2.17) for current, former, and never smokers, respectively. The interaction for family history of diabetes (*P* for interaction = 0.045) did not meet the Bonferroni-corrected significance threshold, and no significant interaction was observed for drinking status (*P* for interaction = 0.218) (Figure 4).

**Figure 4:**
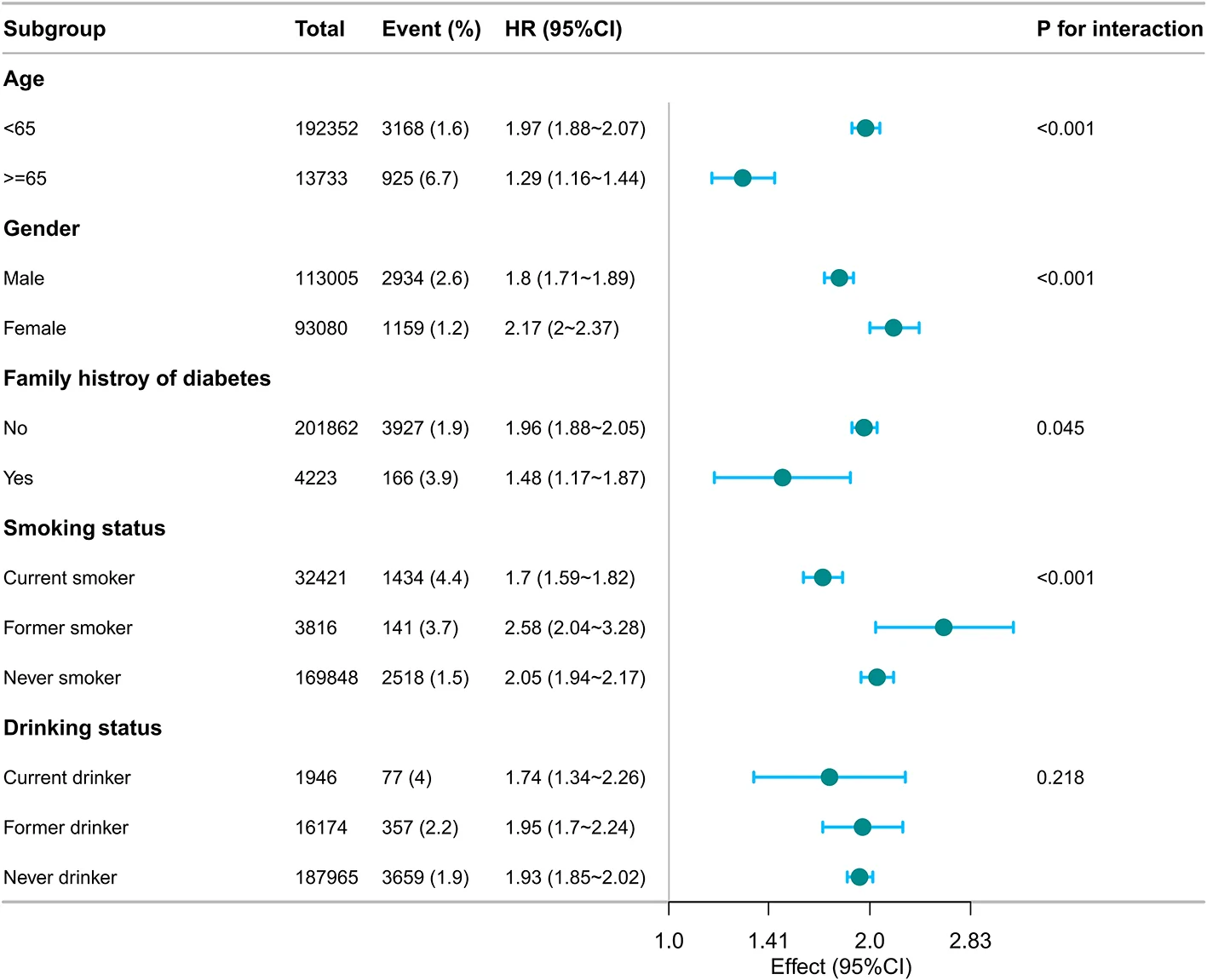
Forest plot of subgroup analyses for ln-TCBI and incident diabetes.

HRs and 95% CIs for incident diabetes per 1-unit increase in ln-TCBI across subgroups. All HRs were adjusted for age, sex, SBP, DBP, family history of diabetes, smoking, drinking, ALT, AST, BUN, and CCR (except for the stratification variable itself). P for interaction: from likelihood ratio tests comparing models with and without interaction terms. TCBI, triglyceride–total cholesterol–body weight index; HR, hazard ratio; CI, confidence interval.

## 4 Discussion

In this large-scale cohort of 206,085 Chinese adults, higher TCBI was significantly associated with an increased risk of incident diabetes. Each 1-unit increase in ln-TCBI was associated with a 94% higher risk, and participants in the highest TCBI quartile had a more than 6-fold higher risk than those in the lowest quartile. A non-linear association and a potential threshold at approximately 923 were observed. Subgroup analyses further indicated heterogeneity in the association across age, sex, and smoking status. These findings extend the existing evidence regarding TCBI by characterizing its association with incident diabetes, including its non-linear pattern, potential threshold, and potential effect modification, in a large cohort of Chinese adults.

The non-linear association between TCBI and incident diabetes may reflect the complex interplay between lipid metabolism, adiposity, insulin resistance, and β-cell dysfunction. Excess adiposity and ectopic lipid accumulation are closely linked to insulin resistance and β-cell dysfunction (22), while obesity-related chronic inflammation may further contribute to metabolic deterioration (23, 24). Similar non-linear or threshold patterns have also been reported for TyG-related indices in relation to diabetes and other metabolic outcomes (25–27). Nevertheless, the threshold of approximately 923 identified in the present study was data-derived rather than a clinically validated cutoff, and its biological and clinical relevance requires further investigation and external validation. Composite nutritional and metabolic indices integrating multiple physiological domains have increasingly been investigated in metabolic and chronic disease research. For example, the prognostic nutritional index has been explored in relation to renal dysfunction and mortality outcomes among patients with diabetes and other chronic diseases (28–30). In cardiovascular risk assessment, TCBI has recently been compared with TyG-BMI (31); however, whether TCBI provides incremental predictive value beyond its individual components or established metabolic indices for incident diabetes remains uncertain.

The stronger association observed among participants aged < 65 years (*P* for interaction < 0.001) is broadly consistent with previous studies reporting age-related heterogeneity in the associations of BMI and TyG-related indices with diabetes risk (18, 26). Younger individuals may exhibit greater metabolic consequences associated with excess adiposity (11). Conversely, the relationship between adiposity and adverse outcomes in older adults may be more complex, as suggested by studies reporting age-related differences in the relationship between adiposity and health outcomes (32, 33). A recent study of the TyG index among individuals with normal fasting glucose and triglyceride levels similarly reported a stronger association with incident diabetes in younger adults (34).

Female sex was also associated with a stronger TCBI–diabetes association. Sex differences in adipose tissue distribution, insulin sensitivity, and β-cell function may contribute to heterogeneity in metabolic risk (35). Experimental and transcriptomic studies have also identified sex-related differences in pancreatic islet biology (36), while epidemiological evidence suggests sex-specific patterns in intermediate hyperglycaemia (37). However, these mechanisms were not directly assessed in the present study, and the observed sex interaction therefore warrants further investigation.

With respect to smoking status, the association between ln-TCBI and incident diabetes was statistically significant across smoking categories, with a numerically higher HR observed among former smokers. Previous studies have reported associations between smoking cessation, post-cessation weight change, and subsequent diabetes risk (38, 39). However, smoking cessation duration and post-cessation weight change were unavailable in the present study; therefore, these mechanisms could not be directly evaluated. This subgroup finding should be interpreted cautiously and requires confirmation in future studies.

Our findings have several potential implications. TCBI is straightforward to calculate and is based on routinely available clinical measurements, suggesting potential utility as an accessible metabolic indicator for epidemiological research. The observed association between TCBI and incident diabetes provides additional epidemiological evidence regarding its association with diabetes risk. However, the present study was designed primarily to characterize the association between TCBI and incident diabetes rather than to establish its superiority over individual metabolic components or other composite indices. Therefore, whether TCBI provides incremental predictive value beyond TG, TC, body weight, or established indices such as TyG-BMI remains to be determined in future comparative studies. Moreover, the threshold of approximately 923 identified in this study was data-derived rather than a clinically validated cutoff and requires further validation in independent populations. The observed heterogeneity across age, sex, and smoking status also warrants confirmation in independent cohorts. More broadly, established lifestyle interventions remain central to diabetes prevention and metabolic risk management (40). Psychosocial factors such as diabetes-related distress are important considerations in the management of individuals with diabetes (41), while healthy dietary patterns may contribute to favorable long-term outcomes among people with diabetes (42).

Several limitations merit consideration. First, the retrospective design precludes causal inference. Second, participants were recruited from individuals undergoing voluntary health examinations and may have been healthier, more health-conscious, and of higher socioeconomic status than the general Chinese population, potentially introducing selection bias and limiting the generalizability of our findings. In particular, the estimated TCBI threshold and subgroup-specific associations may be population-dependent and should be interpreted cautiously. External validation in more representative population-based cohorts is warranted. Third, this study was a secondary analysis of a publicly available dataset, in which incident diabetes status was determined according to the outcome definition of the original study. Separate information distinguishing cases identified by FPG from those identified through self-reported clinician diagnosis was unavailable, precluding an FPG-only sensitivity analysis. Moreover, OGTT and HbA1c measurements were unavailable. Reliance on FPG and self-reported diagnosis alone may therefore have resulted in under-ascertainment or misclassification of incident diabetes, as different glucose-based diagnostic approaches may identify partly different populations (43). Furthermore, differential misclassification cannot be completely excluded, as individuals with higher metabolic risk profiles may have been more likely to undergo glucose testing or receive a diabetes diagnosis. Therefore, the direction and magnitude of potential bias remain uncertain. Fourth, TCBI was assessed only at baseline, and changes in body weight and lipid levels during follow-up were not available. Therefore, a single baseline measurement may not fully reflect long-term TCBI exposure and may have introduced regression dilution bias, potentially attenuating the observed association. Future studies incorporating repeated measurements and time-updated TCBI are needed to evaluate the longitudinal association more accurately.Fifth, residual confounding from unmeasured factors, such as dietary patterns and physical activity, cannot be excluded. Information on smoking cessation duration and post-cessation weight change was also unavailable, limiting further interpretation of the subgroup findings among former smokers.Finally, we were unable to distinguish between type 1 and type 2 diabetes. Although type 2 diabetes accounts for the majority of adult diabetes cases, the lack of diabetes subtype information may have introduced some misclassification.

## 5 Conclusion

Higher TCBI was independently associated with an increased risk of incident diabetes among Chinese adults, with evidence of a non-linear association and a potential threshold at approximately 923. These findings suggest that TCBI may represent an accessible composite metabolic indicator associated with diabetes risk; however, whether TCBI provides additional information beyond established metabolic indicators and whether the identified threshold has clinical relevance require further validation in diverse populations.

## Data Availability

Publicly available datasets were analyzed in this study. This data can be found here. The dataset is publicly available in the Dryad Digital Repository at 10.5061/dryad.ft8750v.
