# Supplementary material for: Association of the triglyceride–cholesterol–body weight index (TCBI) with incident diabetes in Chinese adults: a population-based cohort study with non-linear and threshold analyses
Source: Front Nutr. 2026 Sep 30;13:1928555. doi: 10.3389/fnut.2026.1928555 (PMC13664291; doi:10.3389/fnut.2026.1928555)
Supplement: Supplementary file 1 [file Table_1.docx]

**Supplementary Table 1. Univariate Cox regression for factors associated with incident diabetes**

| Variable | HR (95% CI) | P-value |
| --- | --- | --- |
| Age (years) | 1.07 (1.06–1.07) | <0.001 |
| Gender (female vs. male) | 0.49 (0.46–0.53) | <0.001 |
| Height (cm) | 1.004 (1.000–1.008) | 0.038 |
| Weight (kg) | 1.05 (1.05–1.05) | <0.001 |
| BMI (kg/m²) | 1.24 (1.23–1.24) | <0.001 |
| SBP (mmHg) | 1.04 (1.04–1.04) | <0.001 |
| DBP (mmHg) | 1.05 (1.04–1.05) | <0.001 |
| Baseline FPG (mmol/L) | 10.51 (10.06–10.98) | <0.001 |
| Total cholesterol (mmol/L) | 1.43 (1.39–1.47) | <0.001 |
| Triglyceride (mmol/L) | 1.26 (1.25–1.28) | <0.001 |
| HDL-c (mmol/L) | 0.54 (0.48–0.61) | <0.001 |
| LDL-c (mmol/L) | 1.49 (1.43–1.55) | <0.001 |
| ALT (U/L) | 1.004 (1.004–1.005) | <0.001 |
| AST (U/L) | 1.007 (1.006–1.008) | <0.001 |
| BUN (mmol/L) | 1.25 (1.22–1.27) | <0.001 |
| CCR (umol/L) | 1.006 (1.005–1.007) | <0.001 |
| Final visit FPG (mmol/L) | 1.45 (1.44–1.46) | <0.001 |
| Family history of diabetes (yes vs. no) | 1.71 (1.46–1.99) | <0.001 |
| Drinking status |  |  |
| – Current drinker | 1.00 (Ref) |  |
| – Ever drinker | 0.43 (0.33–0.54) | <0.001 |
| – Never drinker | 0.44 (0.36–0.56) | <0.001 |
| Smoking status |  |  |
| – Current smoker | 1.00 (Ref) |  |
| – Ever smoker | 0.77 (0.65–0.91) | 0.003 |
| – Never smoker | 0.36 (0.34–0.38) | <0.001 |
| TCBI (per 1 SD increase) | 1.15 (1.14–1.15) | <0.001 |
| TCBI.logtrans (cont. var.) | 2.62 (2.53,2.72) | < 0.001 |
| log10TCBI (cont. var.) | 9.21 (8.5,9.98) | < 0.001 |
| TCBI quartiles |  |  |
| – Q1 | 1.00 (Ref) |  |
| – Q2 | 3.07 (2.58–3.63) | <0.001 |
| – Q3 | 6.42 (5.48–7.53) | <0.001 |
| – Q4 | 13.65 (11.71–15.91) | <0.001 |

Notes:HRs and 95% CIs were estimated using univariate Cox models. TCBI was analyzed as a standardized variable (per 1 SD) and as quartiles. Reference groups are indicated for categorical variables. Abbreviations: HR, hazard ratio; CI, confidence interval. For other abbreviations, see Table 1 footnote.
